# Integrated miRNAome, transcriptome, and degradome analyses reveal the role of miRNA–mRNA modules in the biosynthesis of oridonin in *Isodon rubescens*

**DOI:** 10.3389/fpls.2025.1566354

**Published:** 2025-06-18

**Authors:** Lili Zhu, Xiaoxiao Zhang, Hui Guo, Zihan Xu, Zhimin Wang, Liping Dai

**Affiliations:** ^1^ Henan University of Chinese Medicine, Collaborative Innovation Center of Research and Development on the Whole Industry Chain of Yu-Yao, Zhengzhou, Henan, China; ^2^ Engineering Center for Comprehensive Development and Utilization of Authentic Medicinal Materials in Henan Province, Zhengzhou, China; ^3^ Institute of Chinese Materia Medica, China Academy of Chinese Medical Sciences, Beijing, China

**Keywords:** *Isodon rubescens*, miR858b_1ss21GA-MYB module, oridonin biosynthesis, multiomics analysis, dual-luciferase reporter assay

## Abstract

**Introduction:**

*Isodon rubescens* contains many bioactive diterpenoids, especially oridonin, which are used both as medicines and drinks. However, the structure and content of the diterpenoids in *I. rubescens* vary greatly in response to different ecological environments. MicroRNAs (miRNAs) play a pivotal role in the biosynthesis of secondary metabolites; but their roles in *I. rubescens* are obscure.

**Methods:**

This research involved conducting miRNAome, transcriptome, and degradome sequencing analysis of three ecotypes of *I. rubescens*. Furthermore, the regulation of two candidate miRNA–mRNA modules was validated through a dual-luciferase reporter system.

**Results:**

In this study, a total of 1634 miRNAs were identified from 9 miRNAome libraries of three *I. rubescens* ecotypes, which contained various contents of oridonin, lasiodonin, and rosthorin A. Furthermore, 99 DEMs and 8180 DEGs were obtained across three *I. rubescens* ecotypes, and the expressions of selected DEMs and DEGs were verified via reverse transcription quantitative PCR (RT-qPCR). A total of 8928 miRNA-mRNAs networks were identified by degradome analysis, and 23 miRNA-mRNA modules were enriched in the terpenoid biosynthesis pathway. Additionally, 92 negatively correlated DEM‒DEG modules were identified through integrated miRNAome, transcriptome, and degradome analyses, ath-miR858b_1ss21GA‒MYB and ath-miR408-3p_L-1R+1‒*CYP72A219* modules were likely involved in oridonin biosynthesis in *I. rubescens*. Furthermore, the negative regulation of ath-miR858b_1ss21GA targeted MYB was validated through a dual-luciferase reporter system.

**Discussion:**

This study revealed that Ath-miR858b_1ss21GA could repress MYB transcription, potentially downregulating the specific genes involved in the biosynthesis of oridonin and reducing oridonin accumulation in *I. rubescens*.

## Introduction

*Isodon rubescens* (Hemsl.) Hara, which belongs to the genus *Isodon* of Lamiaceae, is a well-known traditional Chinese medicinal herb and healthy herbal tea (Chen et al., 2022). It is also known as Dong Ling Cao in China, and its leaves are generally used for the treatment of sore throat, lumps in the abdomen, or bites caused by insects or snakes, with the purpose of clearing fevers and for detoxification, activating blood, and alleviating pain (Pharmacopoeia, 2020). This herb also has curative effects on many types of cancers, such as throat, esophageal, and breast cancers (Ding et al., 2016; Pi et al., 2017; Gao et al., 2024). Phytochemical studies have shown that this herb contains diterpenoids, phenolic acids, flavonoids, and volatile oils (Xie et al., 2021). Of these compounds, diterpenoids are considered the major bioactive constituents (Sun et al., 2006; Liu et al., 2017). The *ent*-kaurenoid oridonin is the most abundant active component in *I. rubescens* and has been identified as a promising anticancer agent (Li et al., 2021). In recent years, its leaf extracts have been developed into syrups, tablets, granules, and other pharmaceuticals to treat fevers, sore throat, and tonsillitis in China. It is widely distributed in China; however, the structure and the content of the diterpenoids in *I. rubescens* vary greatly under different environmental conditions and have different pharmacological effects and clinical applications, severely restricting the sustainable development of the *I. rubescens* industry (Du et al., 2010; Jin et al., 2015).

Many functional genes involved in oridonin biosynthesis have been identified and verified. The synthesis of oridonin and its derivatives is initiated by the conversion of the general diterpenoid precursor, (*E*,*E*,*E*)-geranylgeranyl diphosphate (GGPP), into various diterpene skeletons by a class II diterpene synthase (diTPS-II) and a class I diTPS (diTPS-I) (Jin et al., 2017; Pelot et al., 2017; Du et al., 2019, 2023). Furthermore, the meristem-specific *IrCYP706V2* and *IrCYP706V7* oxidize the *ent*-kaurene core in the initial stage of oridonin biosynthesis (Sun et al., 2023). Interestingly, alternative splicing alters the diterpenoid outcomes of the class I terpene synthases in *I. rubescens* (Jin et al., 2019). These findings suggest that the posttranscriptional regulation of the terpenoid pathway genes is a fundamental mechanism in oridonin synthesis.

Recently, the posttranscriptional regulation of genes by microRNAs (miRNAs) with lengths of approximately 18–25 nucleotides (nt) has been discovered as a new mechanism involved in plant growth and development, hormone responses, secondary metabolite synthesis, and abiotic and biotic stress responses (Song et al., 2019; Jodder, 2021; Singh et al., 2023). It was found that miRNAs could negatively modulate gene expression by targeting messenger RNAs (mRNAs) for cleavage or by inhibiting their translation based on the complementarity between the miRNA and its targets (Ha and Kim, 2014). The functions of plant miRNAs in the synthesis of secondary metabolites have been extensively explored in medicinal plants (Sun et al., 2022). In *Salvia miltiorrhiza*, miR396b targets *SmGRFs*, *SmHDT1*, and *SmMYB37/4* to regulate cell growth and secondary metabolism, and the overexpression of miR396b in the hairy roots of *S. miltiorrhiza* reduces the concentration of salvianolic acid but enhances the accumulation of tanshinone (Zheng et al., 2020). Investigators have revealed that the high degree of conservation of miR156–SPLs (squamosa promoter binding protein-like genes) in plants plays an important role in the spatiotemporal regulation of sesquiterpene biosynthesis (Yu et al., 2015). In addition, miRNA-initiated reactions involve flavonoids, alkaloids, and other N-containing metabolites (Rongyan et al., 2015). Furthermore, recent research has shown that 27 miRNA–mRNA pairs are involved in the regulation of methyl jasmonate (MeJA) in *I. rubescens* (Lian et al., 2024). Previous research has indicated that MeJA promotes the growth and increases the accumulation of oridonin (Zhang, 2021). These studies suggest that miRNAs play important roles in the synthesis of oridonin in *I. rubescens*.

In this study, we combined the small RNA (sRNA), degradome, and transcriptome sequencing data from *I. rubescens* obtained from different ecological environments to identify potential miRNAs and their targets in *I. rubescens* and to determine their functions in oridonin biosynthesis. Characterized miRNA–mRNA interaction networks could provide valuable information for a better understanding of the molecular regulatory mechanism of oridonin synthesis in *I. rubescens*.

## Materials and methods

### Plant materials and procedures

According to the qualitative evaluations of *I. rubescens* from different places (Chen et al., 2011; Jin et al., 2015), fresh leaves in the vegetative stage were obtained from *I. rubescens* produced in the Taihang Mountains (112.4° E, 35.2° N; Jiyuan city, Henan Province, China, a geo-authentic production area); the Qinling Mountains (113.0° E, 34.7° N; Gongyi city, Henan Province, China, a non-authentic production area); and the Funiu Mountains (112.8° E, 33.7° N; Lushan County, Henan Province, China, a non-authentic production area). Each ecotype included three biological replicates, with each biological replicate derived from the same clonal plant cluster. A portion of the fresh leaves from each biological replicate was cleaned, rapidly frozen in liquid nitrogen, and preserved at −80°C for use in molecular biology experiments. The contents of the main bioactive compounds in the remaining samples were determined via high-performance liquid chromatography (HPLC) according to previously reported methods (Chen et al., 2011). All samples were analyzed with three biological replicates and three technical replicates. The five standard substances, namely, oridonin (Ord, PCS0294), lasiodonin (PCS52081), rosthorin A (PCS2953), rosmarinic acid (PCS0648), and rutin (PCS0724-1), were purchased from HerbSubstance (Chengdu, China), with purities ≥98%.

### Small RNA and mRNA library construction and sequencing

The total RNA of nine samples was extracted using a TRIzol reagent (15596018; Thermo Fisher Scientific, Waltham, MA, USA) following the manufacturer’s instructions. Each ecotype included three biological replicates. The total RNA quantity and purity were measured with a Bioanalyzer 2100 and an RNA 6000 Nano LabChip Kit (5067-1511) (both from Agilent, Santa Clara, CA, USA). The miRNA was ligated to the 3' and 5' RNA adapters and reverse-transcribed to create the first complementary DNA (cDNA) chain. The sRNA sequencing libraries were subsequently prepared using the TruSeq Small RNA Sample Prep Kit (Illumina, San Diego, CA, USA) according to the manufacturer’s standard procedure. The final products were sequenced on an Illumina HiSeq 2000/2500 platform (LC-Bio Technology Co., Ltd., Hangzhou, China) with a result sequencing read length of 1 × 50 bp.

After extraction of the total RNA, the mRNA was purified from the total RNA using Dynabeads Oligo (dT) (Thermo Fisher, Waltham, MA, USA) with two rounds of purification. Following purification, the mRNA was fragmented into short fragments using the Magnesium RNA Fragmentation Module (cat no e6150; NEB, Ipswich, MA, USA). The cleaved RNA fragments were subsequently reverse-transcribed to create cDNA using SuperScript™ II Reverse Transcriptase (cat. no. 1896649; Invitrogen, Carlsbad, CA, USA), which was subsequently used to synthesize U-labeled second-strand DNAs with *Escherichia coli* DNA polymerase I (cat. no. m0209; NEB, Ipswich, MA, USA), RNase H (cat. no. m0297; NEB, Ipswich, MA, USA), and dUTP Solution (cat. no. R0133; Thermo Fisher, Waltham, MA, USA). An A-base was then added to the blunt ends of each strand to prepare them for ligation to the indexed adapters. Each adapter contained a T-base overhang for ligation of the adapter to the A-tailed fragmented DNA. Dual-index adapters were ligated to the fragments, and size selection was performed using AMPureXP beads. After heat-labile UDG enzyme (cat. no m0280; NEB, Ipswich, MA, USA) treatment of the U-labeled second-strand DNAs, the ligated products were amplified with PCR. The average insert size for the final cDNA libraries was 300 ± 50 bp. Finally, 2 × 150-bp paired-end sequencing was performed (PE150) on an Illumina NovaSeq™ 6000 (LC-Bio Technology Co., Ltd., Hangzhou, China) following the manufacturer’s recommended protocol.

### Small RNA and transcriptome sequencing data analysis

The sRNA raw reads were subjected to adapter dimers, junk, and low-complexity removal using ACGT101-miR v4.2 (LC Sciences, Houston, TX, USA). The obtained clean reads with lengths of 18–25 nt were subsequently aligned to the Rfam, Repbase, and mRNA databases to filter the repeats, coding RNA, and other non-coding RNA [e.g., ribosomal RNA (rRNA), transfer RNA (tRNA), small nuclear RNA (snRNA), and small nucleolar RNA (snoRNA)]. These unique sequences were subsequently identified as either known miRNAs or novel 3p- and 5p-derived miRNAs based on a BLAST search using miRBase 22.1 2 (http://www.miRBase.org, accessed in May 2024) (Kozomara et al., 2019). Length variations at both the 3' and 5' ends and one mismatch inside the sequence were allowed. The unique sequences that mapped to specific species of mature miRNAs in hairpin arms were identified as known miRNAs. The unique sequences that mapped to the other arm of the known specific species precursor hairpin opposite to the annotated mature miRNA-containing arm, were considered novel 5p- or 3p-derived miRNA candidates. The remaining sequences that mapped to other selected species precursors (with the exclusion of specific species) in miRBase 22.1 by a BLAST search, or the mapped pre-miRNAs, were further BLASTed against the genomes of specific species to determine their genomic locations, and were both defined as known miRNAs. The unmapped sequences were BLASTed against the specific species genomes, and the hairpin RNA structures containing the sequences were predicted to potentially distinguish novel miRNAs from the flanking 120-nt sequences using the RNAfold software (http://rna.tbi.univie.ac.at/cgi-bin/RNAWebSuite/RNAfold.cgi, accessed in May 2024) based on the criteria for secondary structure prediction (Axtell and Meyers, 2018). The criteria for secondary structure prediction were as follows: 1) number of nucleotides in one bulge in the stem (≤12); 2) number of base pairs in the stem region of the predicted hairpin (≥16); 3) cutoff of free energy (less than or equal to −15 kcal/mol); 4) length of the hairpin (up and down stems + terminal loop ≥50); 5) length of the hairpin loop (≤200); 6) number of nucleotides in one bulge in the mature region (≤4); 7) number of biased errors in one bulge in the mature region (≤2); 8) number of biased bulges in the mature region (≤2); 9) number of errors in the mature region (≤4); 10) number of base pairs in the mature region of the predicted hairpin (≥12); and 11) percentage of mature region in the stem (≥80).

Furthermore, the expression patterns of the miRNAs in the different samples were analyzed using the DESeq package (v 1.12.1) (Anders and Huber, 2010) based on the normalized deep sequencing counts. Transcripts per million (TPM) values were normalized across all samples to enable cross-comparisons, with TPM = (actual miRNA counts/total counts of clean tags) × 10^6^. Differential expression of miRNAs (differentially expressed miRNAs, DEMs) based on normalized deep sequencing counts was analyzed selectively using ANOVA, and the significance threshold was set to 0.05 in each test (*p* < 0.05). The targets of the significantly expressed DEMs were predicted using TargetFinder software. In addition, Gene Ontology (GO) and Kyoto Encyclopedia of Genes and Genomes (KEGG) pathway enrichment analyses of the miRNA targets were annotated, with *p* < 0.05 defined as significantly enriched (Carbon et al., 2021; Kanehisa et al., 2021) using the OmicStudio tools (https://www.omicstudio.cn/tool).

The raw transcriptome reads containing adapters or low-quality bases were removed to obtain high-quality clean reads and were further filtered by Cutadapt with the default parameters (https://cutadapt.readthedocs.io/en/stable/, accessed in May 2024) (Martin, 2011). The clean reads were aligned to the *I. rubescens* genome using the HISAT2 package (version: hisat2-2.2.1; https://daehwankimlab.github.io/hisat2/, accessed in May 2024) (Kim et al., 2019). The mapped reads of each sample were assembled using StringTie (version: stringtie-2.1.6; http://ccb.jhu.edu/software/stringtie/, accessed in May 2024), with the default parameters (Kovaka et al., 2019). The expression levels of all transcripts were determined, and the expression abundance of mRNAs was determined by calculating the fragment per kilobase of transcript per million mapped reads (FPKM) values via StringTie and Ballgown (http://www.bioconductor.org/packages/release/bioc/html/ballgown.html, accessed in May 2024) (Pertea et al., 2016). Differentially expressed genes (DEGs) between the two different groups were analyzed using DESeq2 software (Love et al., 2014). The genes with a false discovery rate (FDR)-adjusted *p*-value (*q*-value) of ≤0.05 and |log2(foldchange)| of ≥1 were considered DEGs. The DEGs were then subjected to enrichment analyses of GO functions and KEGG pathways using the OmicStudio tools (https://www.omicstudio.cn/tool). Among the DEGs, GO terms and pathways meeting these conditions with *p*-values <0.05 were defined as significantly enriched GO terms and KEGG pathways.

### Degradome sequencing analysis and target gene identification

For degradome sequencing, the total RNA from each ecotype was mixed in equal proportions with the total RNA from three biological replicates, and three library constructs were prepared according to the manufacturer’s instructions. The resulting cDNA libraries were analyzed on an Illumina HiSeq 2500 platform (LC Bio, Hangzhou, China) using 50-bp single-end reads. Adapters and low-quality reads were removed from the raw reads to obtain clean reads through a series of data processing steps. The filtered reads were compared with the cDNA database sequences to generate the degraded mRNA fragments. The miRNA–mRNA degradation of potential cleavage sites was subsequently analyzed using the CleaveLand v4.4 pipeline (Addo-Quaye et al., 2009). The cleavage sites were classified into categories 0, 1, 2, 3, and 4. Categories 0–3 had more than one read mapped at the cleavage site with high confidence, while category 4 had only one read mapped with low confidence. Furthermore, Target plots (T-plots) were built to analyze the miRNA targets and the RNA degradation patterns easily using the distributions and the abundance of these transcripts (Addo-Quaye et al., 2008). GO and KEGG analyses of all the identified targets of the DEMs were also performed. Based on the degradome analysis, Pearson’s correlation coefficients for the expression profiles of miRNAs and target genes were also analyzed to identify negative miRNA–mRNA pairs in different comparison groups.

### Validation of the expression patterns via qRT-PCR

We randomly selected eight DEMs and eight DEGs, along with six miRNA–miRNA–target gene pairs, for quantitative real-time PCR (qRT-PCR) analysis across three ecotypes of *I. rubescens*. The total RNA was extracted using a FastPure Universal Plant Total RNA Extraction Kit (Vazyme, Nanjing, China) for mRNA quantification. The quality and purity of the total RNA were measured with a Bioanalyzer 2100 and an RNA 6000 Nano LabChip Kit (5067-1511) (both from Agilent, Santa Clara, CA, USA). The total RNA was subsequently reverse-transcribed using SweScript All-in-One First Strand cDNA Synthesis Super Mix (Servicebio, Wuhan, China) for qRT-PCR. The reaction system was established using the MonAmpTM SYBR^®^ Green qPCR Mix (Monad, Wuhan, China) according to the manufacturer’s instructions and with a Light Cycler 96 instrument (Roche, Shanghai, China). Gene-specific primers were designed using Primer 6.0 (**Supplementary Table S1**). *β-actin* was selected as the reference gene. All RT-qPCRs were performed in triplicate.

For miRNA quantification, sRNAs were isolated using a Plant miRNA Isolation Kit (R6727-00; Omega Bio-Tek, Norcross, GA, USA) according to the manufacturer’s instructions. Reverse transcription products were obtained with a Mir-X miRNA First-Strand Synthesis Kit (638313; Takara, Shiga, Japan). TB Green^®^ Advantage^®^ qPCR Premix (639676; Clontech, San Jose, CA, USA) was used for the qPCR of miRNA according to the manufacturer’s instructions. The universal primer used was reverse-transcribed with U6 as the reference, whereas the miRNA primers in the forward direction were distinct (**Supplementary Table S1**). The reaction protocol consisted of heating at 95°C for 30 s, followed by 40 cycles of heating at 95°C for 10 s, cooling at 60°C for 30 s, and extension at 72°C for 30 s. The relative expression levels of the genes and miRNAs were calculated using the comparative *C*_t_ (2^−ΔΔ^ *^C^* ^t^) method (Livak and Schmittgen, 2001). Values with error bars are presented as the mean of three biological replicates.

### Dual-luciferase reporter assay system

The cDNA was obtained from the leaves in HOr _(from the Taihang Mountains) and used as a template to amplify the precursors of ath-miR858b_1ss21GA, ath-miR408-3p_L-1R+1, and the coding sequence (CDS) region of the targets MYB and CYP72A219. The segment of interest was obtained by reverse transcription PCR (RT-PCR) with specific primers (**Supplementary Table S1**) using Tks Gflex DNA Polymerase (R060A; Takara, Shiga, Japan) following the manufacturer’s instructions. The PCR products were purified, cloned, and then inserted into the pNC-Green-SK plasmid with the Nimble Cloning method using the NovoRec^®^ Plus One-Step PCR Cloning Kit (NR005-01A; Novoprotein, Suzhou, China). The enzyme cleavage sites of the linearized vector were *Bam*HI, *Kpn*I, and *Pst*I. The ligation products were subsequently transformed into *E. coli* DH5α by heat-shock treatment, after which the sequenced recombinant plasmids were transformed into the *Agrobacterium tumefaciens* strain GV3101 (pSoup) as effectors. The sequences of miR858b_1ss21GA and ath-miR408-3p_L-1R+1, which extend 200 bp at each end of the recognition site in the targets MYB and CYP72A219, along with the target sequences, were fused to the firefly luciferase gene on the plasmid PCAMBIA1300-35S-luciferase vector to generate a reporter vector. Each reporter construct was mixed with the effector at a 1:2 ratio and injected into *Nicotiana benthamiana* leaves after 3 h of incubation at room temperature. After incubation for 48 h under low-light conditions, leaf samples were collected. Firefly and Renilla luciferase signals were assayed using a Promega luminescence detector (GloMax 20/20, Promega, Madison, WI, USA) after pretreatment of the leaves using the Dual-Luciferase Reporter Assay System (E1910; Promega, Madison, WI, USA). The LUC/REN ratio was calculated as the relative reporter gene expression level. The Renilla luciferase gene was used as an internal control for the normalization of gene expression.

## Results

### Quantification of five components in *I. rubescens* leaves from three ecotypes

**Figure 1:**
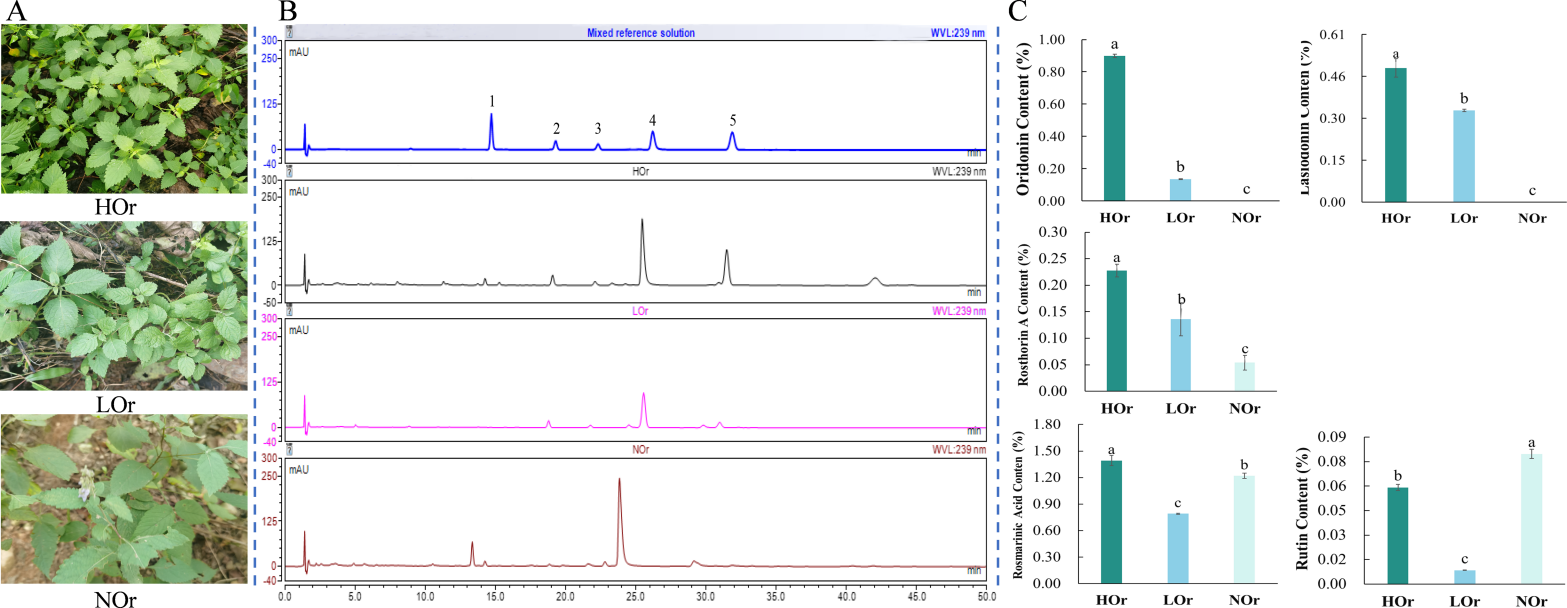
Phenotypic characteristics and analysis of the contents of five components in three *Isodon rubescens* ecotypes. **(A)** Phenotypic characteristics of three *I. rubescens* ecotypes. **(B)** HPLC chromatogram of the mixed reference solution and three *I.rubescens* ecotype samples. Numbers *1–5* represent rutin, lasiodonin, rosthorin A, rosmarinic acid, and oridonin, respectively. **(C)** Contents of the five components of the three *I.rubescens* ecotypes. All data are expressed as the mean standard deviations (*x* ± SD, *n* = 3). *Error bars* indicate SD. One-way ANOVA and the *post-hoc* test were used for multiple testing using SPSS 22.0. A box plot with Tukey’s significance markers **(A–C)** was used to illustrate the group differences. *p* < 0.01.

The contents of the main active components in *I. rubescens* leaves were quantified using HPLC and showed variations across three distinct ecological environments (**Supplementary Table S2** and **Figure 1A**). The contents of three diterpenoids—oridonin, lasiodonin, and rosthorin A—in *I. rubescens* produced in the Taihang Mountains (HOr) were the highest, followed by *I. rubescens* produced in the Qinling Mountains (LOr), while the oridonin content in *I. rubescens* produced in the Funiu Mountains (NOr) was extremely low or absent (**Figures 1B, C**). It is also reported that *I. rubescens* produced in the Funiu Mountains (Lushan County) is a new genotype, named *I. rubescens* f. *lushanensis*, which differs from *I. rubescens* produced in other areas in terms of its plant morphology, chemical composition, and genetic information (Gao Zeng Yi, 1986; Suiqing et al., 2016; Yang et al., 2024). This ultimately diminishes the marketability of *I. rubescens* from non-authentic production areas, making it impossible to sell, and may cause significant economic losses for local farmers. Our study has partially verified the rationale of using genuine production areas of *I. rubescens* that are recorded in the literature (Chen et al., 2011) and laid the foundation for further study.

### Comparative analysis of the transcriptome and miRNAome profiles across three ecotypes of *I. rubescens*

**Figure 2:**
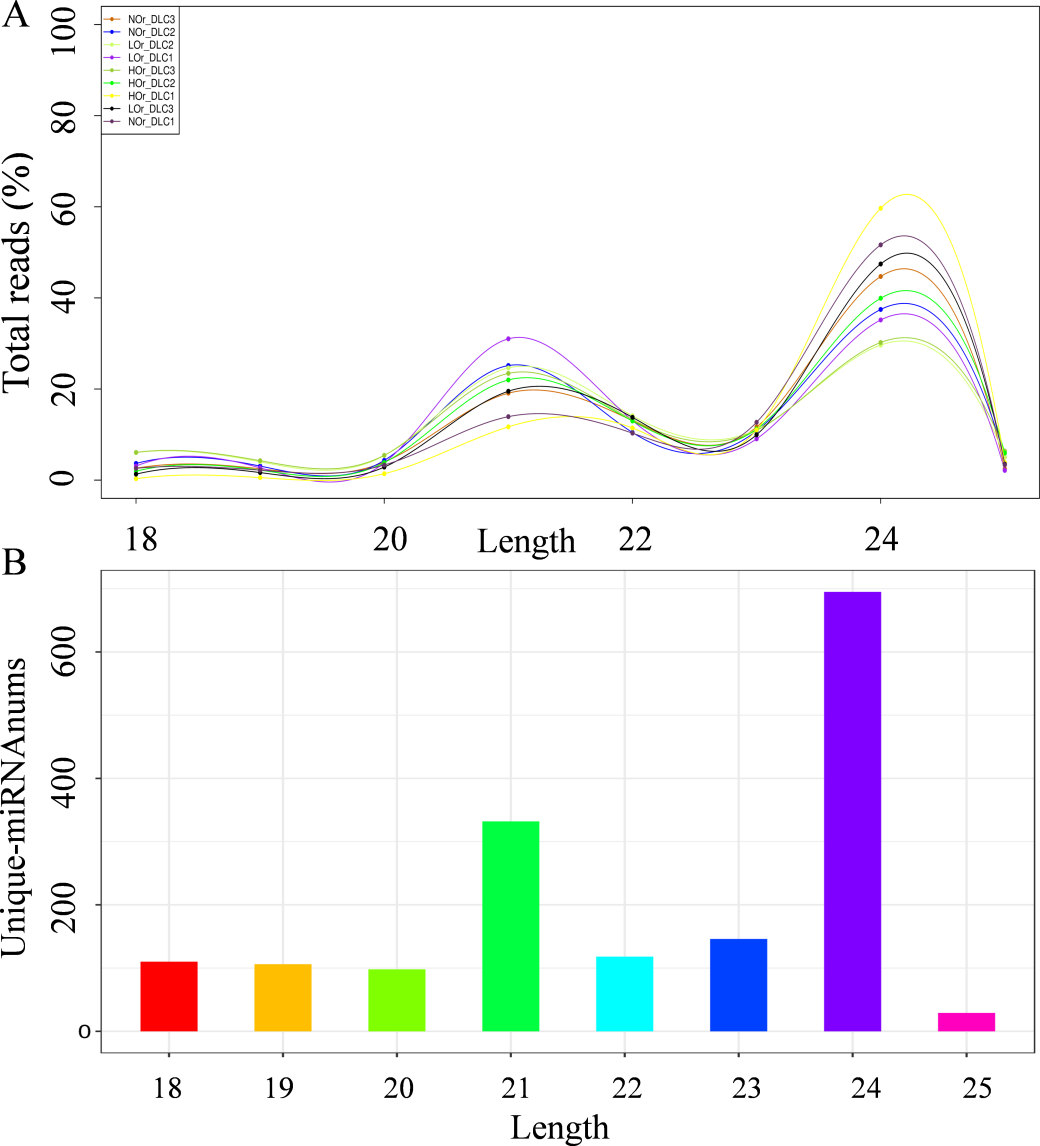
Length distributions of the total reads and unique microRNAs (miRNAs) in *Isodon rubescens*. **(A)** Length distribution of the total reads. **(B)** Number of unique miRNAs of different lengths.

In total, 56.95 Gb of clean reads were obtained from the three *I. rubescens* ecotypes, with an average of 6.33 Gb for each sample, and average Q20 and Q30 values of 98.84% and 94.39%, respectively. In addition, 89.43% of the clean reads were mapped to the *I. rubescens* genome (**Supplementary Table S3**). A total of 113.35 million raw reads and 93.78 Gb of clean reads were generated from the nine sRNA libraries of the three *I. rubescens* ecotypes (**Supplementary Table S4**). On average, the GC content was 51.20%, and the Q30 value of the raw reads was >95.42% for all samples (**Supplementary Table S5**). In addition, a total of 73.07 Gb of valid normalized reads were used for the analysis of sRNAs, which were annotated as rRNA, tRNA, snRNA, and snoRNA. For each library, the annotation results were similar, and rRNA was the most represented, with a mean value of 3.02%, followed by tRNA (0.64%), other Rfam RNAs (0.22%), snRNA (0.06%), and snoRNA (0.03%) (**Supplementary Table S6**). The length distribution of these sRNAs ranged from 18 to 25 nt, of which the 24-nt sRNAs were the most abundant type, followed by the 21-nt sRNAs (**Figure 2A**).

### Identification of known and novel miRNAs in *I. rubescens*

After filtering out the mRNAs, rRNAs, tRNAs, snRNAs, snoRNAs, and other repeats, 509 known miRNAs and 1,125 novel miRNAs were identified. The known miRNAs were classified into four groups (e.g., gp1, gp2a, gp2b, and gp3), with the identified novel miRNAs belonging to group gp4 (**Supplementary Table S6**), according to reported criteria (Ambady et al., 2012). Analysis of the number and length ratios of the unique miRNA sequences revealed that the lengths of the miRNAs were mainly distributed within the 20–24 nt range, and the miRNAs with a length of 24 nt were the most frequent, followed by those with a length of 21 nt (**Figure 2B**). The miRNA sequences were mapped to known miRNAs from 40 plant species, with the highest number of miRNAs mapped to the known gma-miRNAs of *Glycine max* (210, 41.26%), followed by the mdm-miRNAs of *Malus domestica* (183, 35.95%) (**Supplementary Table S8**). Among the identified known miRNA sequences, 325 miRNAs belong to 69 miRNA families. The MIR396 family had the largest number of members (*n* = 21), followed by MIR159 (*n* = 19) and MIR156 (*n* = 18) (**Supplementary Table S9**).

**Figure 3:**
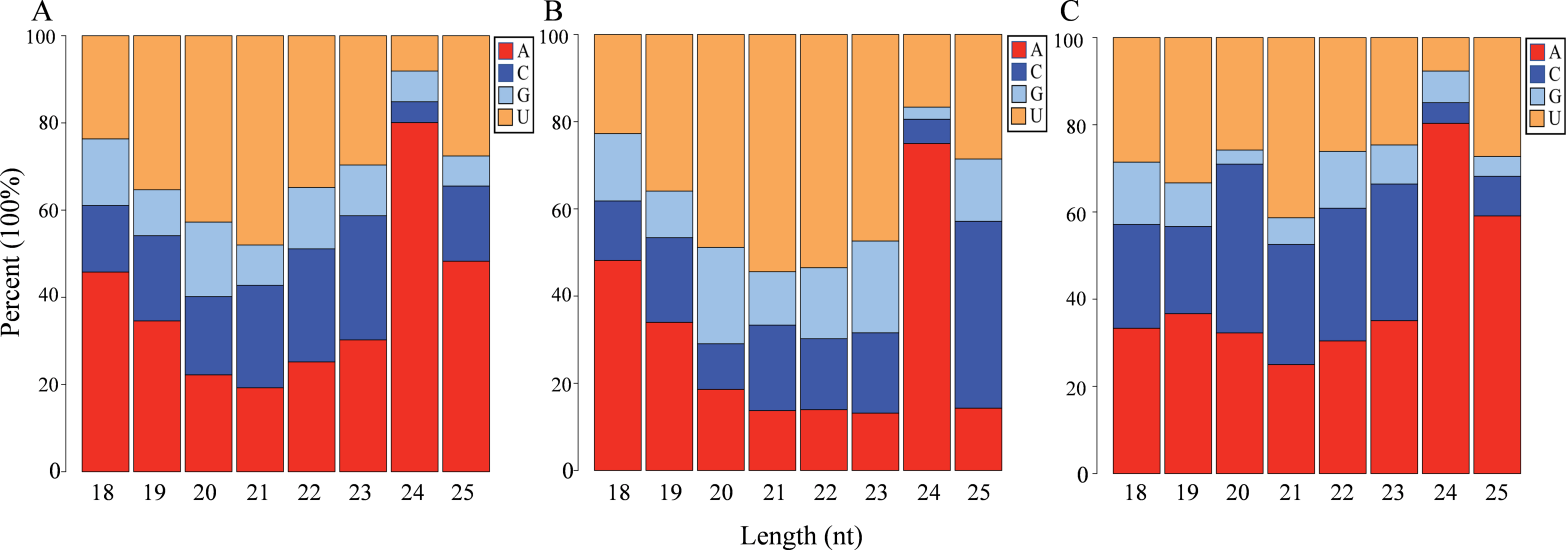
First-nucleotide bias analysis of the microRNAs (miRNAs) in *Isodon rubescens*. **(A–C)** First-nucleotide percentages in total miRNAs **(A)**, known miRNAs **(B)**, and novel miRNAs **(C)**.

Nucleotide bias analysis of the miRNAs in *I. rubescens* showed that the percentages of A, U, C, and G that were distributed in the total miRNAs and novel miRNAs were similar, with A being the most common (30.91% and 32.64%, respectively), followed by U (26.04% and 25.92%, respectively). However, G was most frequently distributed among known miRNAs at 27.67%, followed by A at 26.67% (**Supplementary Table S10**). The miRNA first-nucleotide bias analysis showed that miRNAs of 18 nt and 23–25 nt tended to begin with 5'-A, while the 19- to 22-nt miRNAs tended to start with 5'-U (**Figure 3A**). First-nucleotide bias analysis of the novel miRNAs was different from that known for the miRNAs. Among known miRNAs, those ranging from 20 to 23 nt tended to begin with 5'-U, with 25 nt beginning with 5'-C (**Figure 3B**). However, in the novel miRNAs, 20, 23, and 25 nt tended to begin with 5'-A, with 22 nt beginning with 5'-C (**Figure 3C**).

### Identification of the differentially expressed miRNAs and genes in *I. rubescens* leaves across three ecotypes

**Figure 4:**
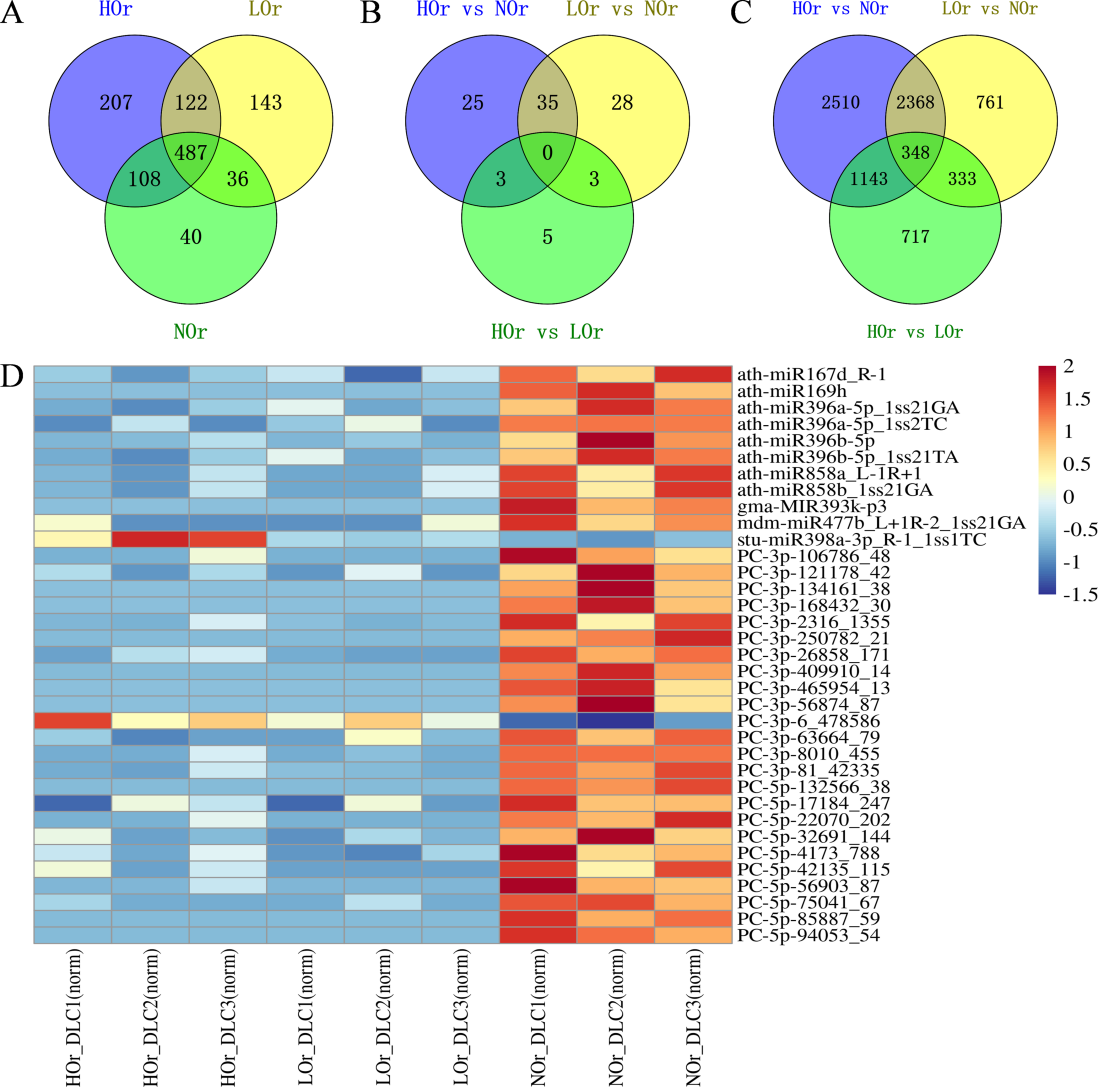
Identification of the differentially expressed miRNAs (DEMs) and genes (DEGs) in three ecotypes of *Isodon rubescens*. **(A)** Identification of miRNAs expressed in three *I. rubescens* ecotypes. **(B, C)** Identification of the DEMs **(B)** and DEGs **(C)** in three *I. rubescens* ecotypes. **(D)** Expression heatmap of the DEMs identified in both HOr *vs.* NOr and LOr *vs.* NOr. The heatmap was generated by the standardized log2(TPM + 0.01). *Red* represents a high expression level, while *blue* represents a low expression level. *HOr*, from the Taihang Mountains; *NOr*, from the Funiu Mountains; *LOr*, from the Qinling Mountains.

We evaluated the miRNA expression in nine libraries from three *I. rubescens* ecotypes. The expression profiles of the identified miRNAs showed that the total expression of most of the detected miRNAs exceeded 10 TPM, approximately 80.72%. It was also found that a total of 1,143 miRNAs had medium or high expression levels (TPM ≥ 10 in at least one sample) in *I. rubescens* leaves (**Supplementary Table S11**). Among them, there were 207, 143, and 40 miRNAs specifically expressed in the HOr, LOr, and NOr samples, respectively (**Figure 4A**). To comprehensively identify the miRNAs involved in the biosynthesis of oridonin in *I. rubescens*, the up- and downregulated miRNAs in the three ecotypes were compared pairwise. A total of 99 DEMs, including 39 known and 60 novel DEMs, were identified across the three comparison groups (**Supplementary Table S12**). In total, 63 and 66 DEMs were detected in HOr *vs.* NOr and LOr *vs.* NOr, respectively (**Figure 4B**). Furthermore, 35 miRNAs, of which 11 were known and 24 were novel, were differentially expressed in these two comparison groups. Interestingly, these 33 DEMs showed a downregulated expression in the HOr and LOr samples compared with the NOr samples (**Figure 4D**). In addition, only 11 DEMs were detected in HOr *vs.* LOr, while csi-miR160c-5p_1ss20TC, cca-MIR156c-p5_2ss7CT17AC, and four novel miRNAs were upregulated in the LOr samples. More importantly, cca-MIR156c-p5 had relatively higher expression levels in the LOr and NOr samples, but was barely expressed in the HOr samples.

**Figure 5:**
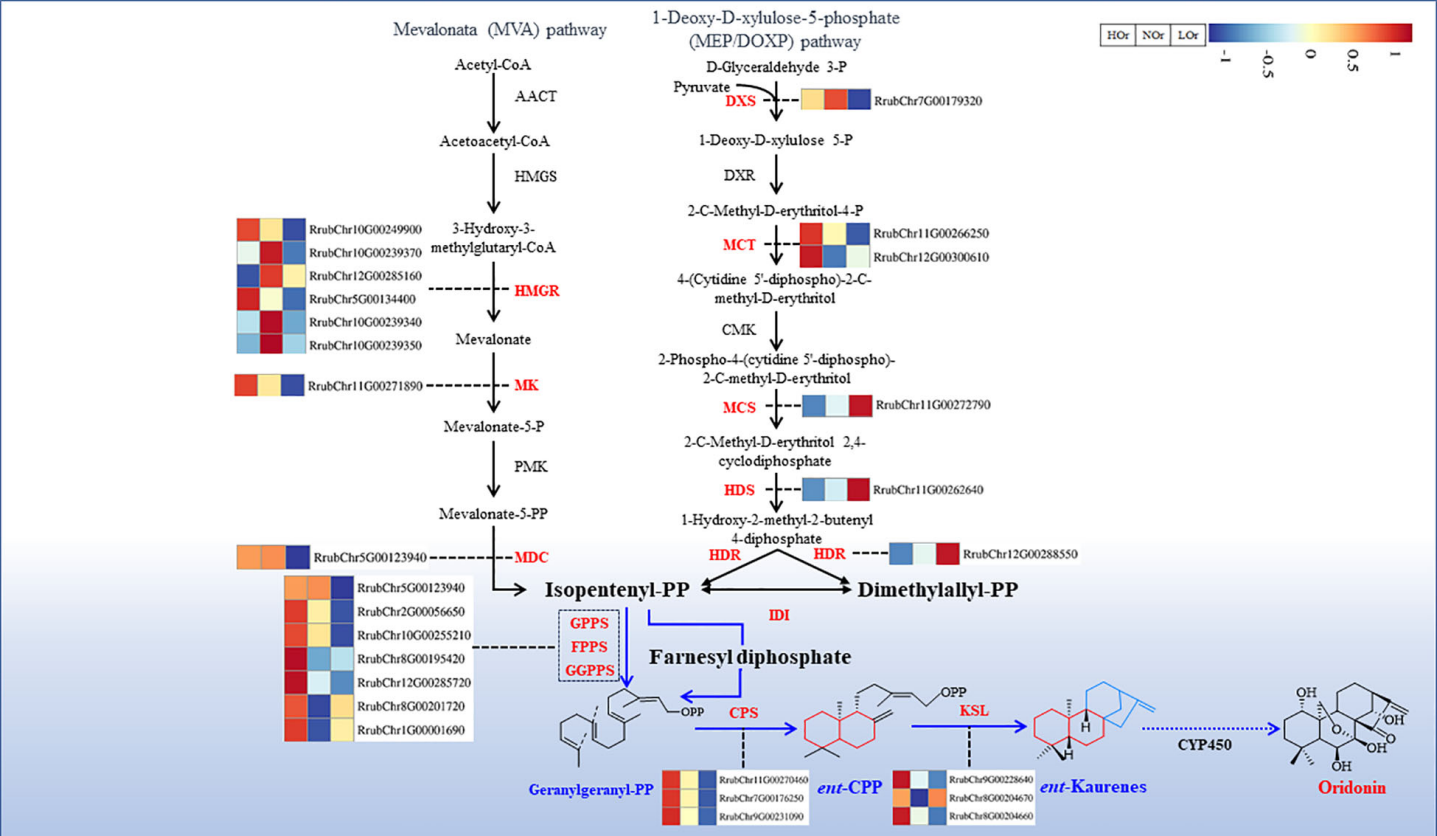
Putative differentially expressed genes (DEGs) involved in oridonin biosynthesis in *Isodon rubescens*. The differential expression heatmap of the DEGs was generated by the standardized log2(average(FPKM) + 0.01). *Red* represents a high expression level, while *blue* represents a low expression level.

Totals of 6,369, 3,810, and 2,541 DEGs were obtained from HOr *vs.* NOr, LOr *vs.* NOr, and HOr *vs.* LOr, respectively (**Figure 4C**). Among all the identified DEGs, 348 significantly expressed DEGs were screened in the three comparison groups. It was found that the majority of DEGs (*n* = 248) were upregulated in the HOr and LOr samples (**Supplementary Table S13** and **Supplementary Figure S1**). In addition, 165 DEGs were significantly upregulated in the HOr samples compared with the LOr or NOr samples and were also upregulated in the LOr samples compared with the NOr samples (**Supplementary Table S13**). Furthermore, KEGG enrichment analysis of all DEGs showed that 62 DEGs were enriched in terpenoid backbone biosynthesis (map00900) or diterpenoid biosynthesis (map00904), and 27 of these DEGs are key enzyme-encoding genes. Interestingly, the expression levels of 24 DEGs were upregulated in the HOr and LOr samples compared with the Lor samples, in particular three CPS genes (**Figure 5**), which were shown to contribute the most to the biosynthesis and accumulation of oridonin in *I. rubescens*.

**Figure 6:**
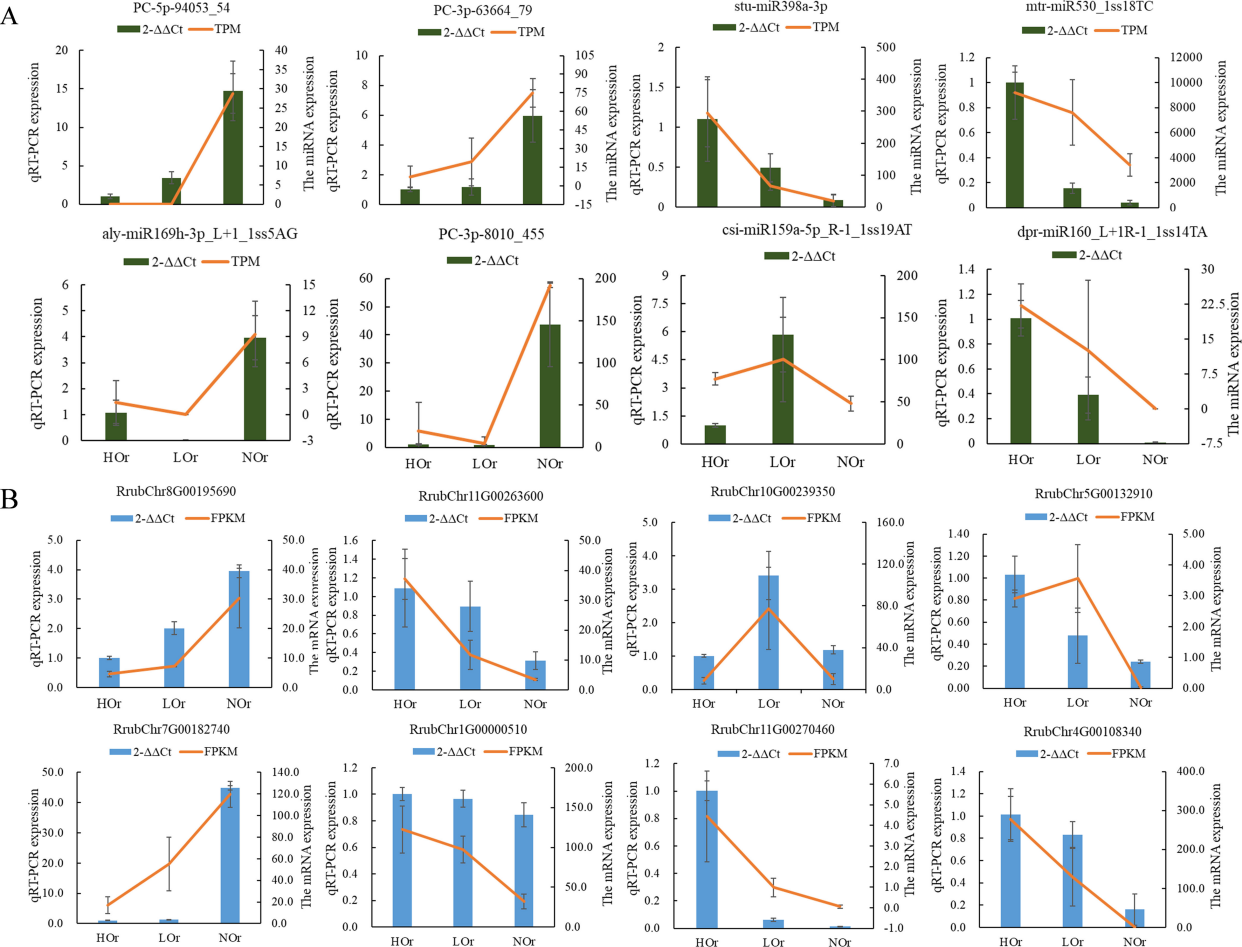
RT-qPCR analysis of the selected differentially expressed microRNAs (DEMs) and genes (DEGs). **(A, B)** Relative expression and transcription levels of the DEMs **(A)** and DEGs **(B)** in *Isodon rubescens*. Three biological replicates were used for each sample, and each biological replicate included three repeated techniques. Relative expression was analyzed with the 2^−ΔΔ^ *^C^* ^t^ method using U6 or the *Actin* gene as the internal reference for miRNA or mRNA, respectively.

To validate the accuracy and reliability of the mRNA and miRNA expression profiling results, RT-qPCR analysis was conducted to determine the expression patterns of several DEMs and DEGs. Their specific primers were designed using Primer Premier 6. The expression trends of eight miRNAs and eight mRNAs agreed well with their TPM and FPKM obtained from the sRNA and RNA sequencing (RNA-seq) data, respectively. The results confirmed the accuracy of the identification of DEMs and DEGs in this study (**Figures 6A, B**).

### Target prediction and degradome analyses

In plants, miRNAs generally anti-regulate gene expression mainly by cleaving targeted mRNAs. The targets of *I. rubescens* miRNAs were predicted using the psRNATarget server (http://plantgrn.noble.org/psRNATarget/). A total of 73 DEMs containing 33 known DEMs and 537 target genes formed 706 miRNA–target mRNA pairs with co-expression in nine samples were identified (**Supplementary Table S14**). These DEM targets were predominantly plant–pathogen interaction proteins, plant hormone signal transduction proteins, growth-regulating factors, and transcription factors (TFs). Importantly, a number of targets involved in terpenoid biosynthesis were also obtained, such as FPS (farnesyl pyrophosphate synthase 1), cytochrome P450 (CYP450) CYP76AH8, CYP76AH16, 78A5, CYP82D47-like, and CYP450 94B3-like.

**Figure 7:**
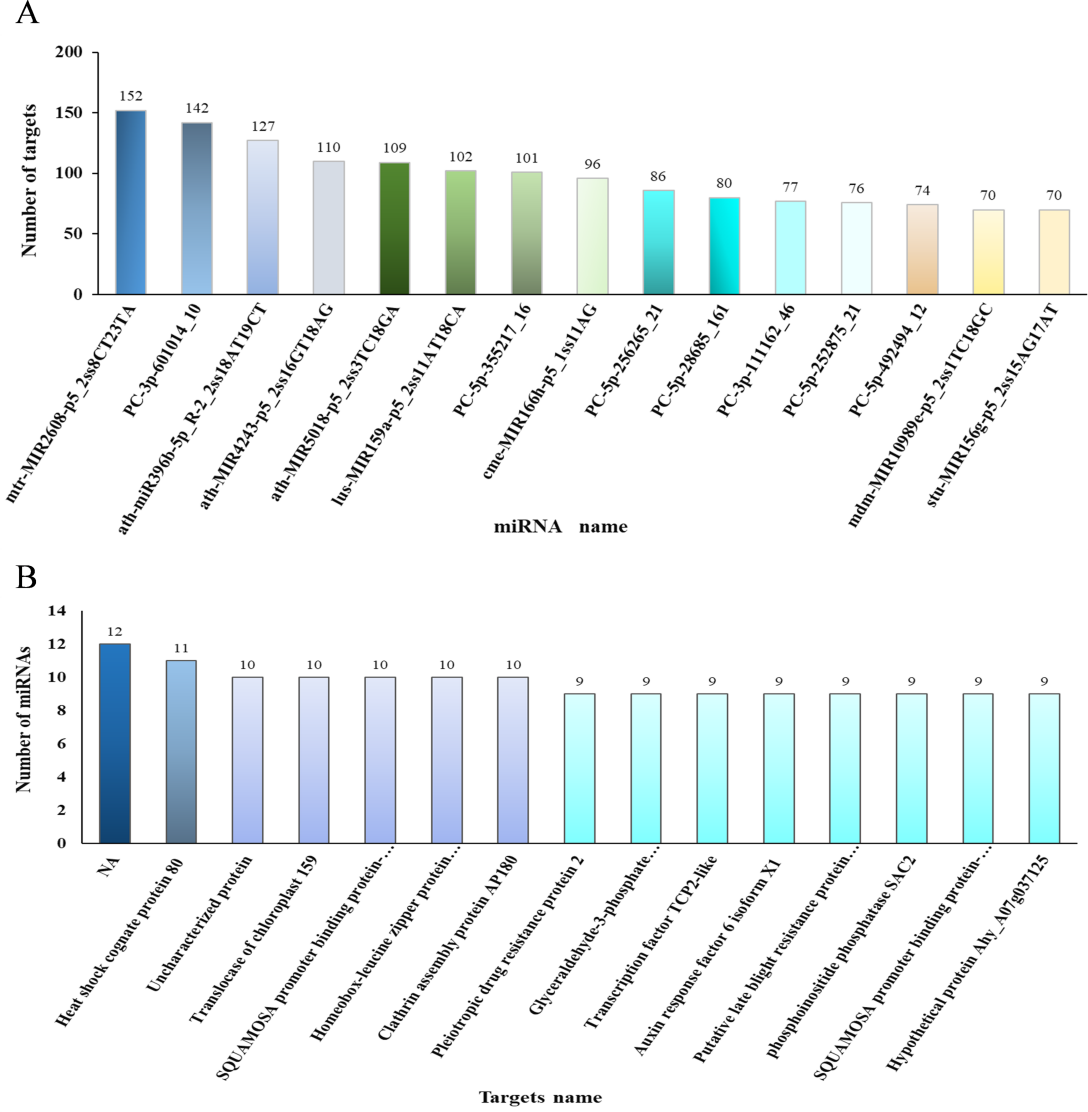
Number analysis of the targets and microRNAs (miRNAs). **(A)** Target numbers of the top 15 miRNAs based on degradome analysis. **(B)** Number of the top 15 miRNA targets based on degradome analysis, where *NA* represents an undefined protein.

Three degradome pools were also analyzed and sequenced to identify the miRNA targets. Degradome sequencing assays of the HOr, LOr, and NOr samples generated 10,391,127, 10,913,877, and 20,447,067 raw sequence reads, respectively, of which 5,863,449, 6,051,441, and 6,556,943, respectively, were unique (**Supplementary Table S15**). The clean tags were mapped to the reference database of the *I. rubescens* transcriptome sequence. A total of 70,455 (76.65%) covered transcripts of the 91,920 input transcripts were determined. In total, 785 miRNAs and 5,392 target degradome transcripts that could form 8,928 regulatory pairs were obtained in the sequencing of three degradome pools (**Supplementary Table S16**). These results imply that a single miRNA could target more than one target gene; for example, mtr-MIR2608-p5_2ss8CT23TA targeted the largest number of predicted target genes, with 152 target genes identified, followed by the novel miRNA PC-3p-601014_10 (142 target genes) and ath-miR396b-5p_R-2_2ss18AT19CT (127 target genes) (**Figure 7A**). Furthermore, more than one miRNA was found to be able to target in the same transcript; for example, 12 miRNAs simultaneously targeted RrubChr11G00274200.1 (unknown), followed by RrubChr12G00297340.1 (11 miRNAs; heat shock cognate protein 80, HSP80) and RrubChr6G00157110.1 (10 miRNAs; SQUAMOSA promoter binding protein-like 5, SPL5) (**Figure 7B**). Previous studies revealed that the miRNA-induced regulatory effects could be propagated through TFs, and it was found that some of the miRNA target genes were also annotated as TFs, including *SPL*, *MYB*, *ARF*, *bHLH*, *WRKY*, and *GRF* (**Supplementary Table S16**).

### GO enrichment and KEGG pathway analyses of three degradome transcripts

To better understand the functions of miRNAs, GO and KEGG enrichment analyses were performed based on the identified miRNA degradome transcripts. A total of 4,004 degradome transcripts from the HOr *vs.* NOr group were enriched in 3,284 GO_Terms, including 1,793 biological processes, 400 cellular components, and 1,091 molecular functions (**Supplementary Table S17**), with 401 GO_Terms being significantly enriched (*p* < 0.05). Furthermore, the top 20 most abundant GO terms, including cytoplasm (GO:0005737), chloroplast (GO:0009507), protein binding (GO:0005515), cytosol (GO:0005829), the Golgi apparatus (GO:0005794), and other biological processes, were related to photosynthesis and abiotic stress (**Supplementary Figure S2A**). In addition, 3,841 and 4,271 degradome transcripts from the LOr *vs.* NOr and HOr *vs.* LOr groups were enriched in 3,255 and 3,437 GO_Terms, respectively (**Supplementary Table S17**). Interestingly, the GO enrichments of the degradome transcripts from the three comparison groups were similar, and the top 20 most abundant GO terms were almost the same (**Supplementary Figures S2B, C**).

**Figure 8:**
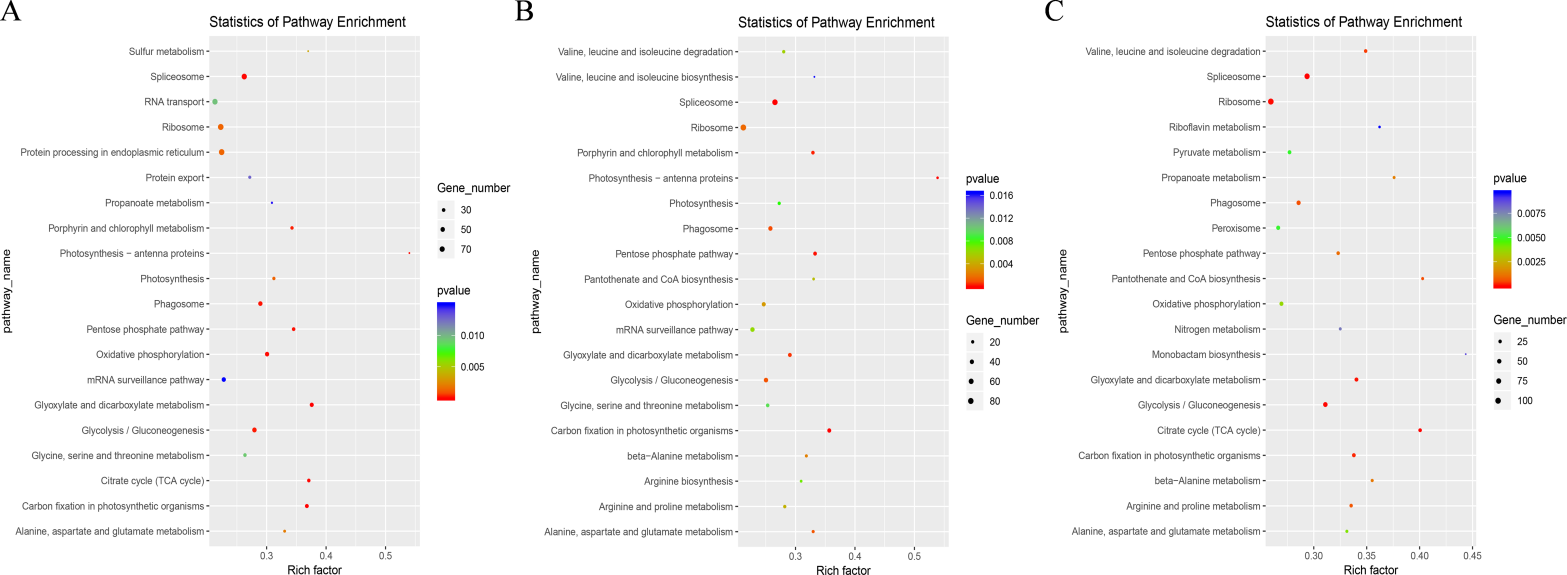
Top 20 Kyoto Encyclopedia of Genes and Genomes (KEGG) pathways in three comparison groups based on degradome transcripts. **(A)** Top 20 KEGG pathways in the HOr *vs.* NOr group. **(B)** Top 20 KEGG pathways in the LOr *vs.* NOr group. **(C)** The top 20 KEGG pathways in the HOr *vs.* LOr group. *HOr*, produced in the Taihang Mountains; *NOr*, produced in the Funiu Mountains; *LOr*, produced in the Qinling Mountains.

In addition, the top 20 enriched KEGG pathways of the degradome transcripts from the three comparison groups were also similar (**Figures 8A–C**), and nine pathways were the same, including “spliceosome (ko03040),” “ribosome (ko03010),” “carbon fixation in photosynthetic organisms (ko00710),” “glycolysis/gluconeogenesis (ko00010),” “phagosome (ko04145),” and other pathways related to genetic information regulation and transduction and carbon metabolism. The degradome transcripts were also enriched in “plant hormone signal transduction (ko04075),” “zeatin biosynthesis (ko00908),” “brassinosteroid biosynthesis (ko00905),” and “indole alkaloid biosynthesis (ko00901), in addition to other plant hormone biosynthesis (**Supplementary Table S18**). Importantly, the degradome transcripts were enriched in terpenes, flavonoids, and other active ingredient biosynthetic pathways, such as terpenoid backbone biosynthesis (ko00900), monoterpenoid biosynthesis (ko00902), diterpenoid biosynthesis (ko00904), sesquiterpenoid and triterpenoid biosynthesis (ko00909), and flavone and flavonol biosynthesis (ko00944). These results suggest that miRNAs could modulate gene expression by targeting mRNAs and that they play a key role in the dynamic accumulation of terpenes and the synthesis of flavonoids in *I. rubescens* from different types of ecological environments.

### Putative miRNA–mRNA modules involved in oridonin biosynthesis in *I. rubescens*

**Figure 9:**
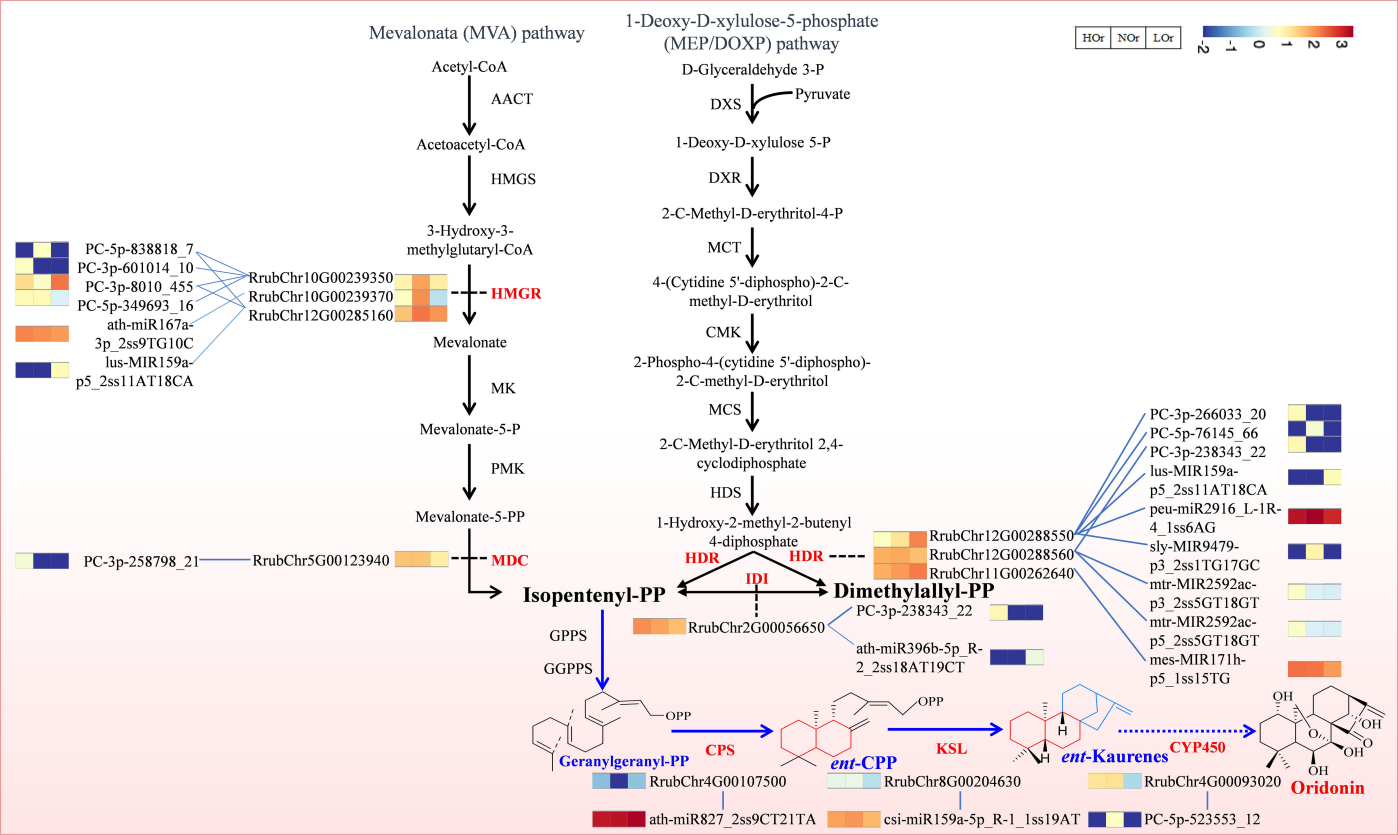
Putative microRNA (miRNA)–messenger RNA (mRNA) modules involved in oridonin biosynthesis in *Isodon rubescens* based on three degradome transcripts. The differential expression heatmaps of the miRNAs and target mRNAs were generated by the standardized log2(average(TPM) + 0.01) or log2(average(FPKM) + 0.01). *Red* represents high expression levels, while *blue* represents low expression levels.

A total of 32 miRNAs and 23 genes that could form 86 regulatory pairs were found to be associated with the terpenoid backbone biosynthesis (ko00900) and diterpenoid biosynthesis (ko00904) pathways based on three degradome transcripts (**Supplementary Table S19**). According to the gene annotations, a total of 23 miRNA–mRNA modules composed of 19 miRNAs, of which 11 are known and eight are novel miRNAs, and 11 target genes encoding seven enzymes were the most likely directly involved in oridonin biosynthesis (**Figure 9**). Among them, ath-miR167a-3p_2ss9TG10C, lus-MIR159a-p5_2ss11AT18CA, and four novel miRNAs all targeted HMGR (3-hydroxy-3-methylglutaryl coenzyme A reductase), which is the rate-limiting step in the mevalonate (MVA) pathway. PC-3p-258798_21 targeted MDC (mevalonate pyrophosphate decarboxylase), which catalyzes the last step of the MVA pathway. In addition, lus-MIR159a-p5_2ss11AT18CA, peu-miR2916_L-1R-4_1ss6AG, sly-MIR9479-p3_2ss1TG17GC, mtr-MIR2592ac-p3_2ss5GT18GT, mtr-MIR2592ac-p5_2ss5GT18GT, mes-MIR171h-p5_1ss15TG, and three novel miRNAs all targeted HDR, which catalyzes the last step of the methylerythritol phosphate (MEP) pathway. PC-3p-238343_22 and ath-miR396b-5p_R-2_2ss18AT19CT both targeted IDI (isopentenyl diphosphate isomerase), which controls the availability and isomerization of IPP (isopentenyl diphosphate) and DMAPP (dimethylallyl diphosphate), which are essential in the overall biosynthesis of all terpenoids. Furthermore, ath-miR827_2ss9CT21TA, csi-miR159a-5p_R-1_1ss19AT, and PC-5p-523553_12 targeted CPS (copalyl diphosphate synthase), KSL (kaurene synthase-like), and CYP450, respectively, and they are directly involved in terpenoid skeleton formation and the oxidation-reduction reactions of *ent*-kaurene in the initial stage of oridonin biosynthesis. Taking advantage of the available transcriptomics data for the same samples, we also observed that the expression of the majority of miRNAs and their target genes presented opposing patterns, which is consistent with the phenomenon of miRNAs negatively controlling their target genes. It is thus speculated that these miRNAs, including lus-MIR159a-p5_2ss11AT18CA, ath-miR827_2ss9CT21T, and csi-miR159a-5p_R-1_1ss19AT, which noticeably presented opposite expression patterns compared with their target genes, may play essential regulatory roles in the biosynthesis of oridonin.

### Spearman’s correlation analysis of the miRNAs and mRNAs

**Figure 10:**
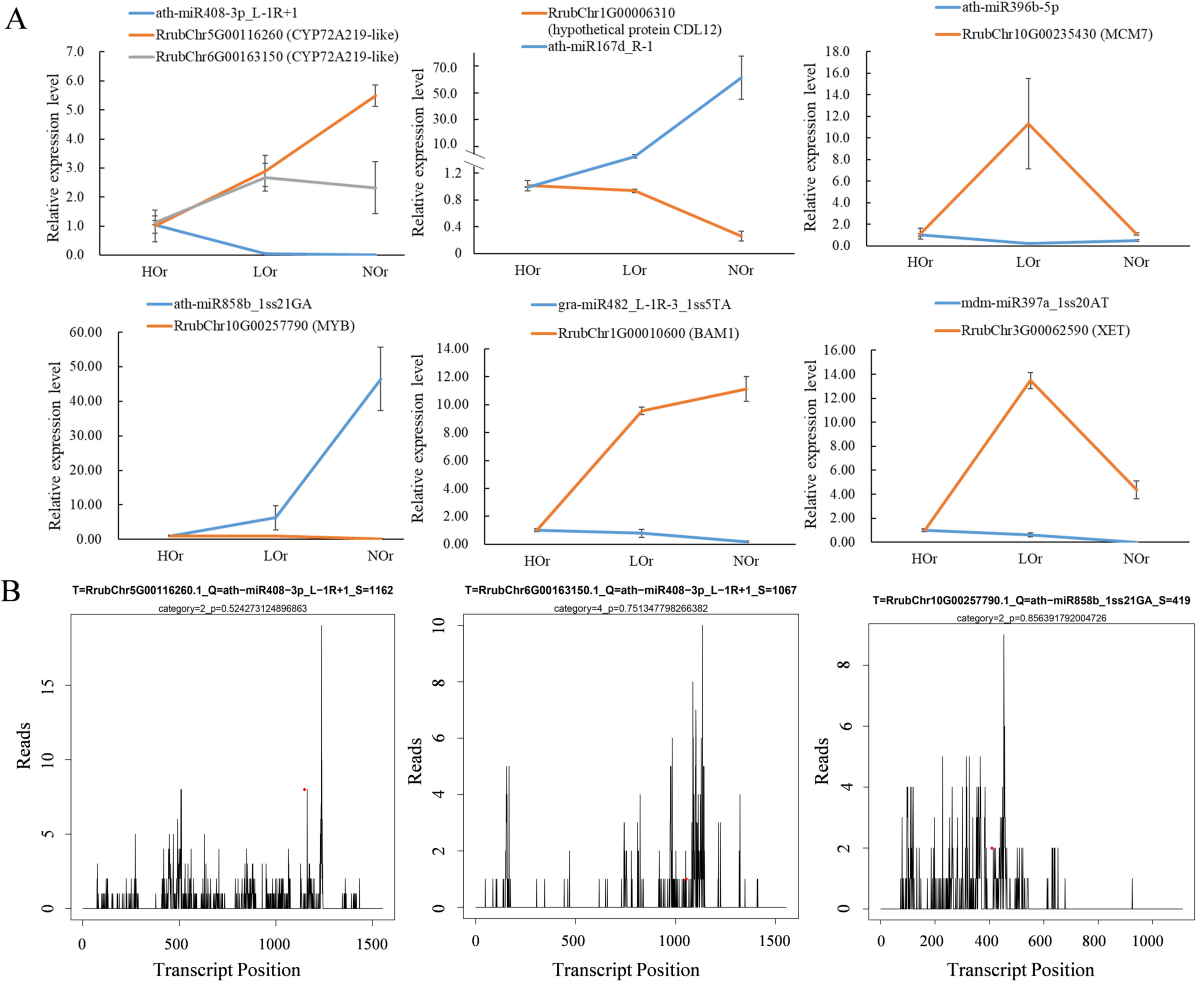
Expression trends and nucleotide cleavage sites of the microRNAs (miRNAs) and negative targets in *Isodon rubescens*. **(A)** Expression trends of the miRNAs and negative targets in *I. rubescens* based on quantitative real-time PCR (qRT-PCR) analysis. **(B)** Target plots (T-plots) of the miRNA targets confirmed by degradome sequencing. In the T-plots, *red dots* indicate the miRNA-directed cleaved transcripts. The *X*-axis indicates the nucleotide position in the target complementary DNA (cDNA). The *Y*-axis indicates the number of reads of cleaved transcripts detected in the degradome cDNA library.

Negatively correlated DEM and targeted DEG modules were identified through Pearson’s correlation analysis. A total of 92 modules, including 69, 28, and 7 negatively correlated DEMs–target DEGs, were identified in the three comparison groups (**Supplementary Table S20**). A total of nine DEM–target DEG modules were found in both HOr *vs.* NOr and LOr *vs.* NOr, which contained seven DEMs and seven DEGs. Among them, ath-miR167d_R-1, ath-miR396a-5p_1ss2TC, ath-miR396b-5p, and ath-miR396b-5p_1ss21TA all negatively targeted the hypothetical protein CDL12, PC-3p-2316_1355, and mitogen-activated protein kinase 2 (MAP2K2); ath-miR858b_1ss21GA negatively targeted the MYB transcription factor; and stu-miR398a-3p_R-1_1ss1TC negatively targeted two proteins, cucumber peeling cupredoxin and primary amine oxidase. In addition, ath-miR408-3p_L-1R+1 negatively targeted two CYP450 CYP72A219-like transcripts that were obtained in HOr *vs.* NOr and HOr *vs.* LOr. Importantly, the results of the RT-qPCR also indicated that the expression of several miRNAs and their target genes presented opposing expression patterns (**Figure 10A**), which is consistent with the general phenomenon of miRNAs negatively regulating genes. Furthermore, the efficacy of this miRNA-mediated cleavage of target genes was predicted using the T-plots through degradation. The degradation categories of the two CYP72A219-like transcripts mediated by ath-miR408-3p_L-1R+1 varied, for example, Rrubclr5G00116260 category = 2 and RrubChr6G00163150 category = 4 (**Figure 10B**). Moreover, the category value of ath-miR858b_1ss21GA, which targeted RrubChr10G00257790 (MYB), was 2, suggesting that the module had more than one read mapped at the cleavage sites with high confidence.

### Verification of the interaction between miRNAs and target genes

**Figure 11:**
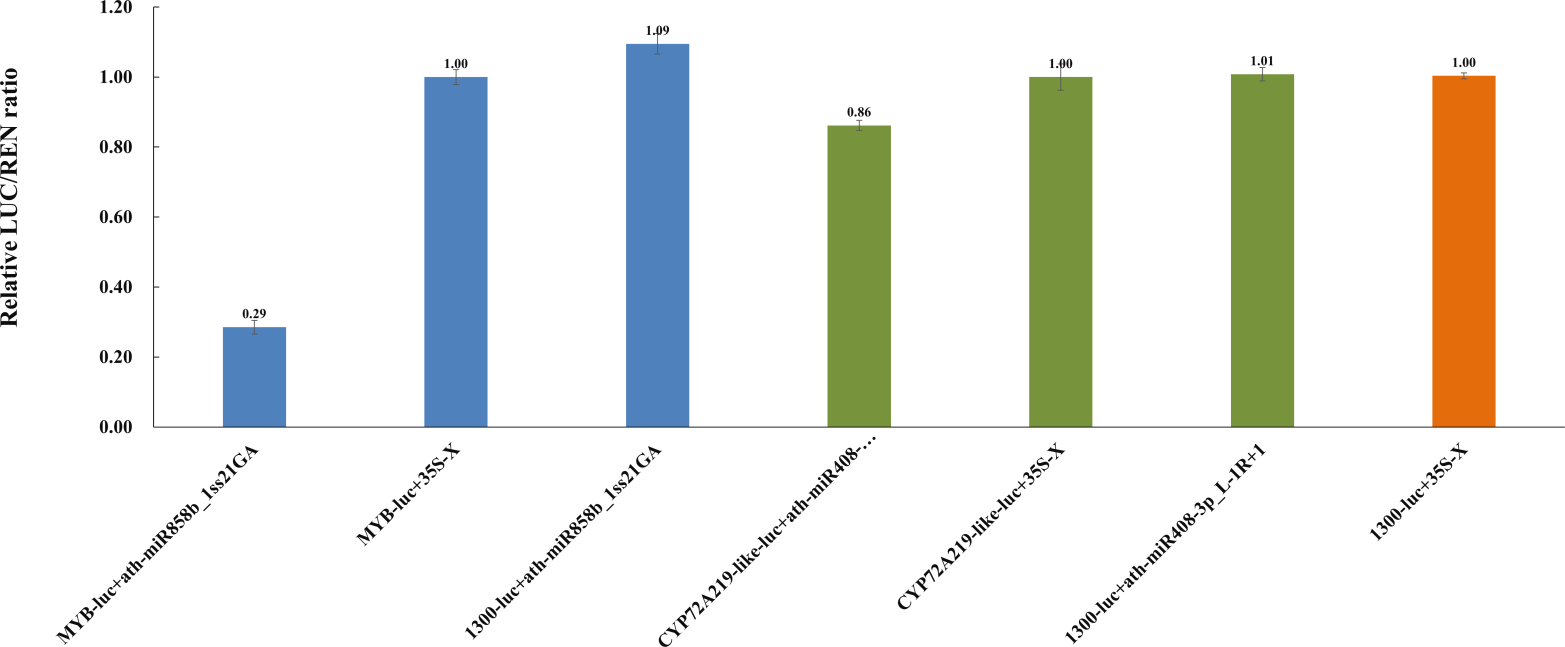
Relative LUC/REN ratios in ath-miR858b_1ss21GA+MYB and ath-miR408-3p_L-1R+1+CYP72A219-like.

To validate the interactions between miRNAs and the target genes, a dual-luciferase reporter assay was carried out in *N. benthamiana* leaves. Compared with the control group lacking ath-miR858b_1ss21GA or *MYB*, the LUC/REN ratio noticeably decreased and was more than four time lower after the co-transformation of miR858b with the target sequence *MYB* (**Figure 11** and **Supplementary Table S21**). The results show that ath-miR858b_1ss21GA could be combined with the target sequence and that it decreased the transcriptional activity of *MYB*. In contrast, the LUC/REN ratio decreased insignificantly after the co-transformation of ath-miR408-3p_L-1R+1 and *CYP72A219-like* compared with the control group that lacked ath-miR408-3p_L-1R+1 or CYP72A219-like. This suggests that ath-miR408-3p_L-1R+1 is less likely to bind to the target sequence *CYP72A219-like*.

## Discussion

Terpenoids are essential for interaction with the environment and for the provision of defense mechanisms in plants, such as antimicrobial, antifungal, antiviral, and anti-parasitic properties (Tholl, 2015; Ali et al., 2022). *I. rubescens* is a typical perennial medicinal herb with a variety of diterpenoids, which are the most structurally and quantitatively diverse components and which exhibit a wide variety of pharmacological activities, such as oridonin, rubescensine B, and enmein (Liu et al., 2017; Zhang et al., 2017; He et al., 2018; Chen et al., 2022). Our results also indicate that the composition and contents of diterpenoids in *I. rubescens* are strongly dependent on the local environment, which is consistent with previous reports (Han et al., 2003; Zhang et al., 2010; Xie et al., 2021). One of the important epigenetic factors, miRNAs, are usually non-coding RNAs that are 21–24 nt in length, which can regulate the expression of the structural genes and TFs on the terpene biosynthetic pathway at the posttranscriptional levels through splicing and blocking translation, and controlling the biosynthesis of terpenes in medicinal plants (Sun et al., 2022). Although a previous study was conducted to explore the molecular mechanism of miRNA in *I. rubescens* in response to MeJA (Lian et al., 2024), the mechanism of the posttranscriptional regulation of miRNAs involved in oridonin biosynthesis is still not well understood. In this study, we conducted deep sequencing of the sRNA, transcriptome, and degradome of *I. rubescens* obtained from three geographical regions to explore the potential mechanisms of the miRNAs involved in diterpenoid biosynthesis.

### MiRNAs regulate plant phenotypic plasticity triggered by environmental stimuli

Our research shows that oridonin, lasiodonin, rosthorin, rosmarinic acid, and rutin were all significantly differentially distributed in the three *I. rubescens* ecotypes. A total of 1,634 miRNAs representing 69 miRNA families were identified, of which 99 miRNAs were differentially expressed across the three *I. rubescens* ecotypes (**Figure 4B**). As reported, miRNAs are involved in the regulation of plant phenotypic plasticity triggered by light, temperature, nutrients, and other environmental stimuli (Song et al., 2019). In *Arabidopsis*, miR396 can be induced to repress its target *GROWTH REGULATING FACTORs* (*GRFs*), thereby mediating the inhibition of leaf growth in response to UV-B radiation (Casadevall et al., 2013). MiR156 regulates the flowering time in *Arabidopsis* under low temperatures by targeting a subset of *SPL* genes (Lee et al., 2010). Cold-inducible miR393 also downregulates the TEOSINTE BRANCHED1, CYCLOIDEA, and PROLIFERATING CELL NUCLEAR ANTIGEN BINDING FACTOR genes and enhances cold tolerance in rice (Yang et al., 2013). In this study, 33 DEMs were downregulated in the HOr and LOr samples compared with the NOr samples. Of those downregulated, 10 are known miRNAs: ath-miR396a-5p_1ss21GA, ath-miR396b-5p_1ss21TA, ath-miR396a-5p_1ss2TC, ath-miR167d_R-1, ath-miR169h, gma-MIR393k-p3, mdm-miR477b_L+1R-2_1ss21GA, ath-miR858a_L-1R+1, ath-miR858b_1ss21GA, and ath-miR396b-5p (**Figure 4D**). MiRNAs typically negatively regulate gene expression and participate in various secondary metabolic processes in plants, such as terpenoids, flavonoids, anthocyanidins, and phenolic acids, among others (Li et al., 2024). Hence, it was speculated that these DEMs are most likely to deactivate their target genes involved in oridonin biosynthesis in response to different ecological environments.

### MiRNA–mRNA regulatory networks are involved in oridonin biosynthesis

Analysis of the degradome transcripts revealed 23 interaction modules that may participate in the biosynthesis of oridonin (**Figure 9**). Among them, PC-3p-8010_455 had a higher expression in the NOr samples compared with that in the HOr and LOr samples, and it targeted the rate-limiting enzyme gene in the MVA pathway, *HMGR*, which can directly affect terpenoid products in *Arabidopsis thaliana* (Ohyama et al., 2007). In *Persicaria minor*, the upregulation of pmi-miR6300 could lead to the suppression of *HMGR*, while its downregulation could lead to the high accumulation of *HMGR*, a potential genetic tool for the regulation of terpenoid biosynthesis in *P. minor* induced by *Fusarium oxysporum* (Samad et al., 2019). In addition, ath-miR396b-5p_R-2_2ss18AT19CT, ath-miR827_2ss9CT21T, and csi-miR159a-5p_R-1_1ss19AT targeted *IDI*, CPS, and *KSL*, respectively. These targets are directly involved in terpenoid skeleton formation and oxidation–reduction reactions. Furthermore, these three miRNAs all had obvious opposing expressions compared with their targets that are involved in the biosynthesis of oridonin. In *Paeonia lactiflora* Pall., miR396, miR393, miR835, miR1144, miR3638, miR5794, and miR9555 have been verified as monoterpenoid biosynthesis-related miRNAs through degradome sequencing (Lian et al., 2024). It has also been reported that miR156 and miR160 could target *DISPL* and *DfARF*, respectively, to regulate terpenoid biosynthesis in *Dryopteris fragrans* (Song et al., 2022). More research showed that a variety of different miRNAs, including miR390, miR156, miR845b, miR1134, and miR854, have been identified to be associated with terpenoid biosynthesis (Kajal and Singh, 2017; Xu et al., 2023; Lian et al., 2024).

### MiRNA–TF networks for oridonin biosynthesis in *I. rubescens*

Research has indicated that a number of TFs participate in terpenoid biosynthesis by binding to specific DNA sequences of the target genes, such as AP2/ERF, bHLH, WRKY, MYB, and SPL (Huang et al., 2024). In this study, many target genes of miR156, miR858, miR159, miR160, miR396, miR172, and other miRNA families were also linked to SPL, MYB, ARF, bHLH, WRKY, and GRF (**Supplementary Table S16**). Recently, several studies have demonstrated that the miR156, miR396, and miR858 family members are linked to genes encoding SPL, MYB, and WRKY transcription factors. The miR156-SPL module plays an important role in the spatiotemporal regulation of sesquiterpene biosynthesis in *A. thaliana* and *Pogostemon cablin*, as SPL directly binds to the TPS promoter and activates its expression (Yu et al., 2015). The overexpression of miR396b in Danshen hairy roots inhibited hairy root growth and reduced the salvianolic acid concentration, but enhanced the tanshinone accumulation through targeting SmGRFs, SmHDT1, and SmMYB37/4 (Zheng et al., 2020). In *I. rubescens*, it was shown that MeJA stimulated the accumulation of oridonin, and mtr-miR396b-5p_1ss21GA-GRF9, ath-miR858a_L-1R+1-MYB114, mtr-miR159a-MYB80, csi-miR159a-3p_R+3-MYB4, ssl-miR156_R-1-SPL6, mtr-miR156b-5p_L+1-SPL5, and gma-MIR5368-p3_1ss2GA-ARF5 were found to be linked to MeJA regulation and oridonin biosynthesis through degradome sequencing (Lian et al., 2024). In summary, many miRNAs and TFs could work together to construct miRNA–TF networks for oridonin metabolite synthesis in *I. rubescens.*

### Negatively correlated DEM–DEG regulatory modules in *I. rubescens*

Spearman’s correlation analysis of the miRNAs and mRNAs showed that nine DEM–DEG negative regulation modules, including PC-3p-2316_1355–MAP2K2 and ath-miR858b_1ss21GA–MYB, were found in both the HOr *vs.* NOr and LOr *vs.* NOr groups, and the ath-miR408-3p_L-1R+1–CYP450 CYP72A219-like module was found in both the HOr *vs.* NOr and HOr *vs.* LOr groups (**Figure 10A**). It was also reported that MYB proteins interact with the promoters of the genes encoding the biosynthetic enzyme TPS, which is associated with terpenoid biosynthesis in plants(Ke et al., 2021). In addition, CYP450 CYP706V oxidase genes could control oridonin biosynthesis in the shoot apex (Sun et al., 2023). More importantly, miRNAs could regulate MYB and CYP450 involved in the biosynthesis of bioactive compounds in plants. In *Sapindus mukorossi*, multiple miRNA–mRNA regulatory networks including ath-miR5021–SmIDI2/SmGPS5/SmbAS1/SmCYP71D-3/SmUGT74G-2, han-miR3630-3p–SmCYP71A-14/SmbHLH54/SmMYB135/SmWRKY32, and ppe-miR858–SmMYB5/SmMYB32 were found to be potentially involved in triterpenoid saponin biosynthesis through integrative analysis of the miRNAs and mRNAs (Xu et al., 2023). In *Hylocereus monacanthus*, Hmo-miR157b–HmSPL6-like, Hmo-miR6020–HmCYP71A8-like, Hmo-novel-2–HmCYP83B1-like, Hmo-novel-15–HmTPST-like, Hmo-miR858-HmMYB12-like, Hmo-miR858–HmMYBC1-like, and Hmo-miR858–HmMYB2-like were verified by 5'-RACE and a transient expression system in tobacco and may play important roles in pitaya fruit coloration and betalain accumulation (Chen et al., 2020). Thus, it is speculated that the miR858b_1ss21GA–MYB and ath-miR408-3p_L-1R+1–CYP72A219 modules are most likely involved in the biosynthesis of oridonin in *I. rubescens*.

### Ath-miR858b_1ss21GA negatively regulates the *MYB* gene involved in oridonin biosynthesis

**Figure 12:**
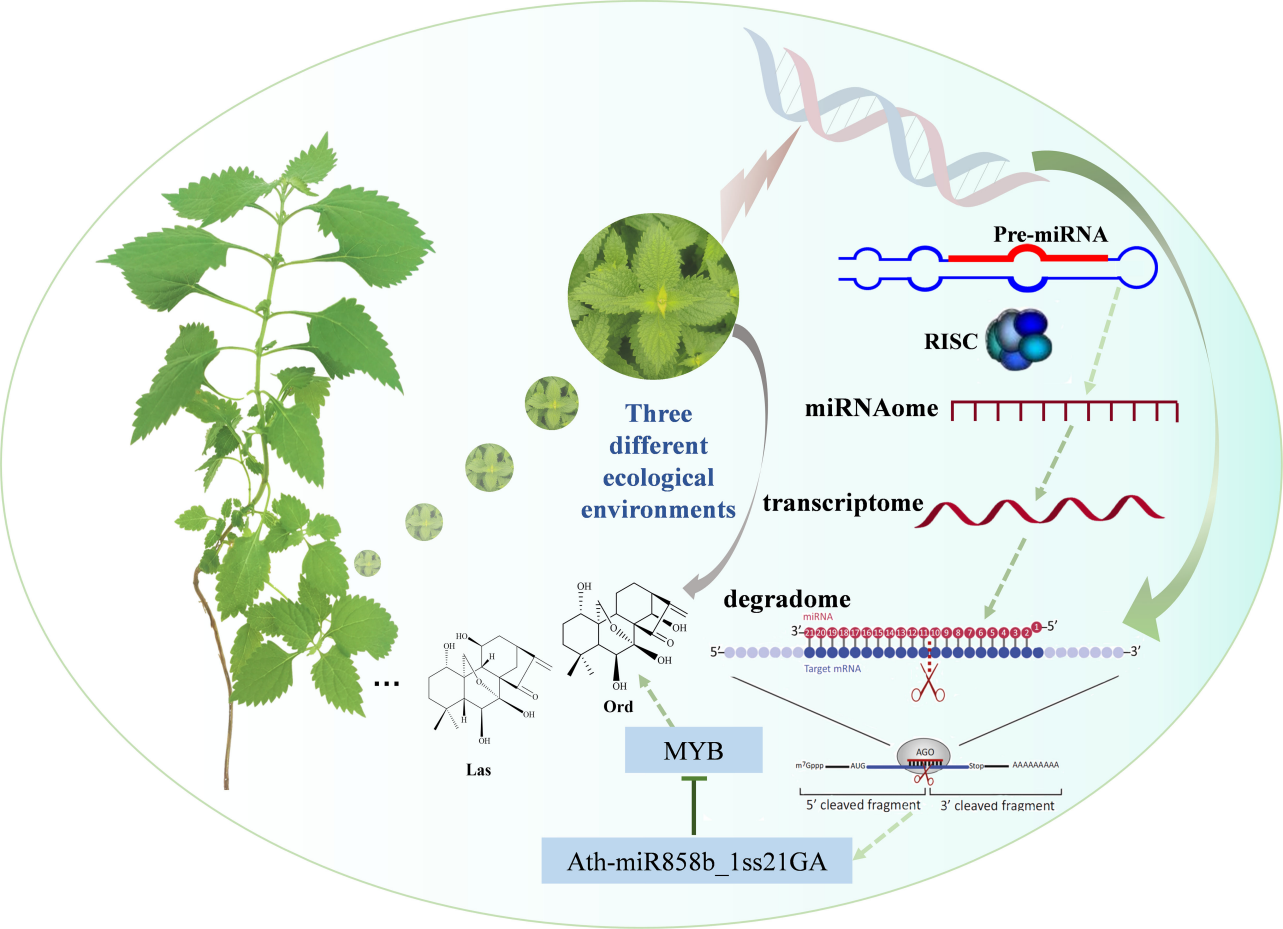
Multi-omics analysis uncovers the microRNA (miRNA)–messenger RNA (mRNA) networks that regulate oridonin biosynthesis in *Isodon rubescens*.

The results of the dual-luciferase reporter assay indicate that ath-miR858b_1ss21GA could combine with a target sequence and decrease the transcriptional activity of MYB; however, ath-miR408-3p_L-1R+1 is less likely to decrease the transcriptional activity of its target sequence CYP72A219-like (**Figure 11**). Previous studies have suggested that miR858 can target the *MYB* transcription factor, which can affect a range of secondary metabolic processes, including proanthocyanidin, flavonol, and terpenoids (Wang et al., 2024). In *S. miltiorrhiza*, a well-known traditional Chinese medicine, the overexpression of Smi-miR858a caused significant growth retardation and tanshinone and phenolic acid reductions, while the Smi-miR858a-SmMYB module could activate the expression of *SmPAL1* and *SmTAT1*, which are involved in phenolic acid biosynthesis, and *SmCPS1* and *SmKSL1*, which are associated with tanshinone biosynthesis (Zhu et al., 2024). Analysis of the phylogenetic relationship of *I. rubescens* and *S. miltiorrhiza* indicated that they share a close genetic relationship, which is due to their shared belonging to the Lamiaceae family of medicinal plants (Sun et al., 2023). Numerous reports have shown that MYBs could act as crucial regulators of the genes encoding biosynthetic enzymes involved in terpenoids, such as *GPPS* and *TPS*, to regulate the production of terpenoids (Bhatt et al., 2025). Ath-miR858b_1ss21GA potentially represses the expression of MYB, thereby downregulating the oridonin biosynthesis genes and reducing the oridonin accumulation in *I. rubescens* (**Figure 12**).

## Conclusions

In this study, a total of 509 known and 1,125 novel miRNAs were identified in *I. rubescens*. Among them, 99 miRNAs were differentially expressed across three *I. rubescens* ecotypes. Most of the identified DEMs were found to be downregulated in the HOr and LOr samples compared with the NOr samples. At the same time, 348 DEGs were screened in the three comparison groups, with 248 DEGs found to be upregulated in the HOr and LOr samples compared with the NOr samples. The expression patterns of these DEMs and mRNAs were analyzed in various samples using RT-qPCR. There were 8,928 miRNA–mRNA networks that included 785 miRNAs and 5,392 targets identified by degradome analysis. Furthermore, 23 miRNA–mRNA modules were enriched in the terpenoid biosynthesis pathway. In addition, many targets of miR156, miR858, miR159, miR160, miR396, and miR172 were found to be linked to SPL, MYB, ARF, bHLH, WRKY, and GRF. A total of 92 negatively correlated DEM–DEG modules were identified through integrated miRNAome and transcriptome analyses, and a preliminary RT-qPCR verification supported the negative regulation pattern. Notably, the results of the dual-luciferase assay provided direct evidence that ath-miR858b_1ss21GA suppresses the transcription of MYB. These results provide molecular evidence revealing the comprehensive miRNA–mRNA regulatory networks that participate in the biosynthesis of oridonin in *I. rubescens*.

## Data Availability

The original contributions presented in the study are included in the article and the Supplementary Material. The transcriptome raw data presented in this study have been submitted to NCBI (Accession number is PRJNA1214546, SRR32137346 - SRR32137354), small RNA raw data (SRR32135299 - SRR32137347), and the degradome raw data (SRR32175898 - SRR32175900).
