# Supplementary figures and images for: Integrated miRNAome, transcriptome, and degradome analyses reveal the role of miRNA–mRNA modules in the biosynthesis of oridonin in Isodon rubescens
Source: Front Plant Sci. 2025 Jun 18;16:1566354. doi: 10.3389/fpls.2025.1566354 (PMC12213554; doi:10.3389/fpls.2025.1566354)

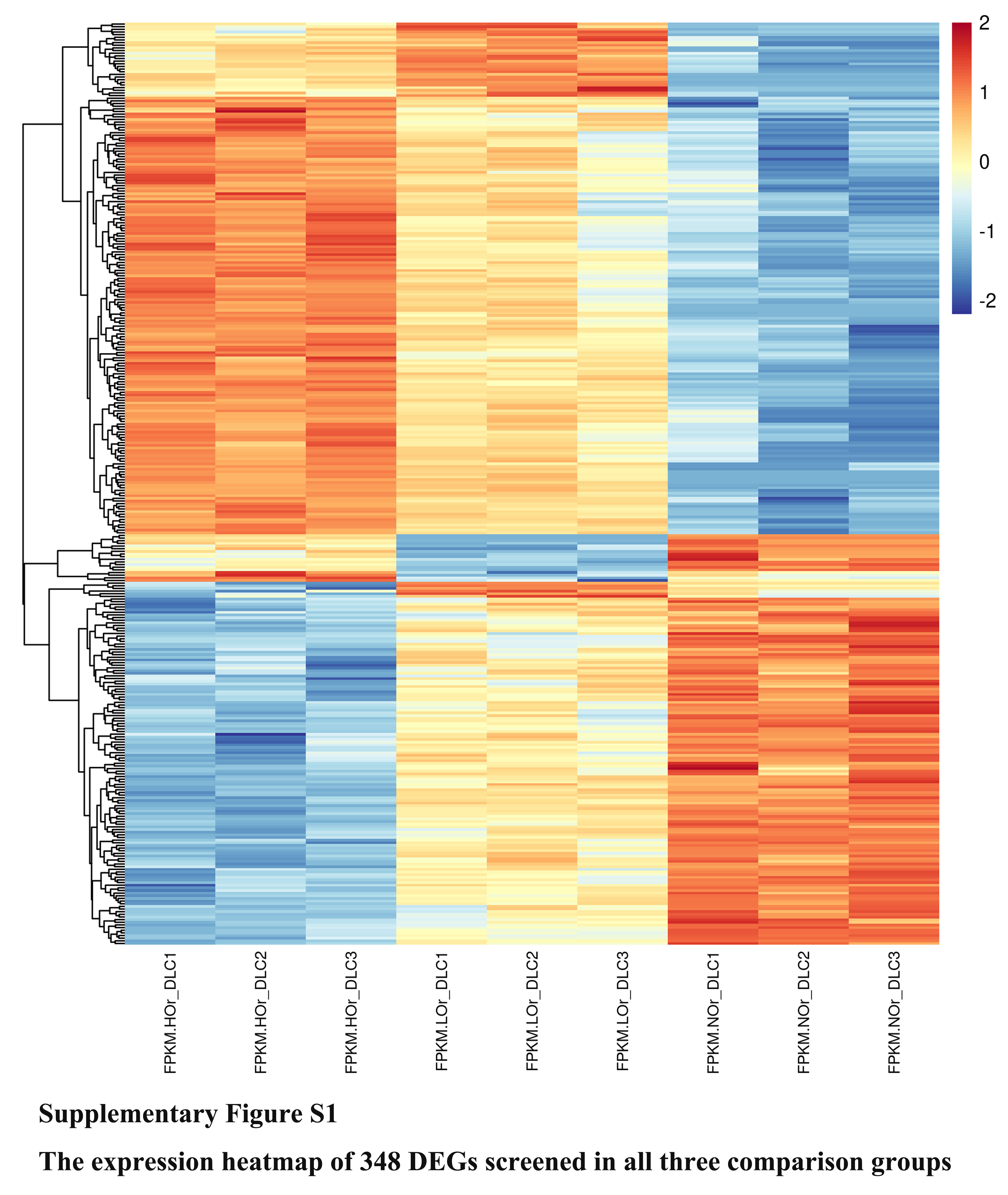

Supplement: Supplementary file 1 [file DataSheet1.zip › Supplementary Material/Supplementary Figure S1.tif]

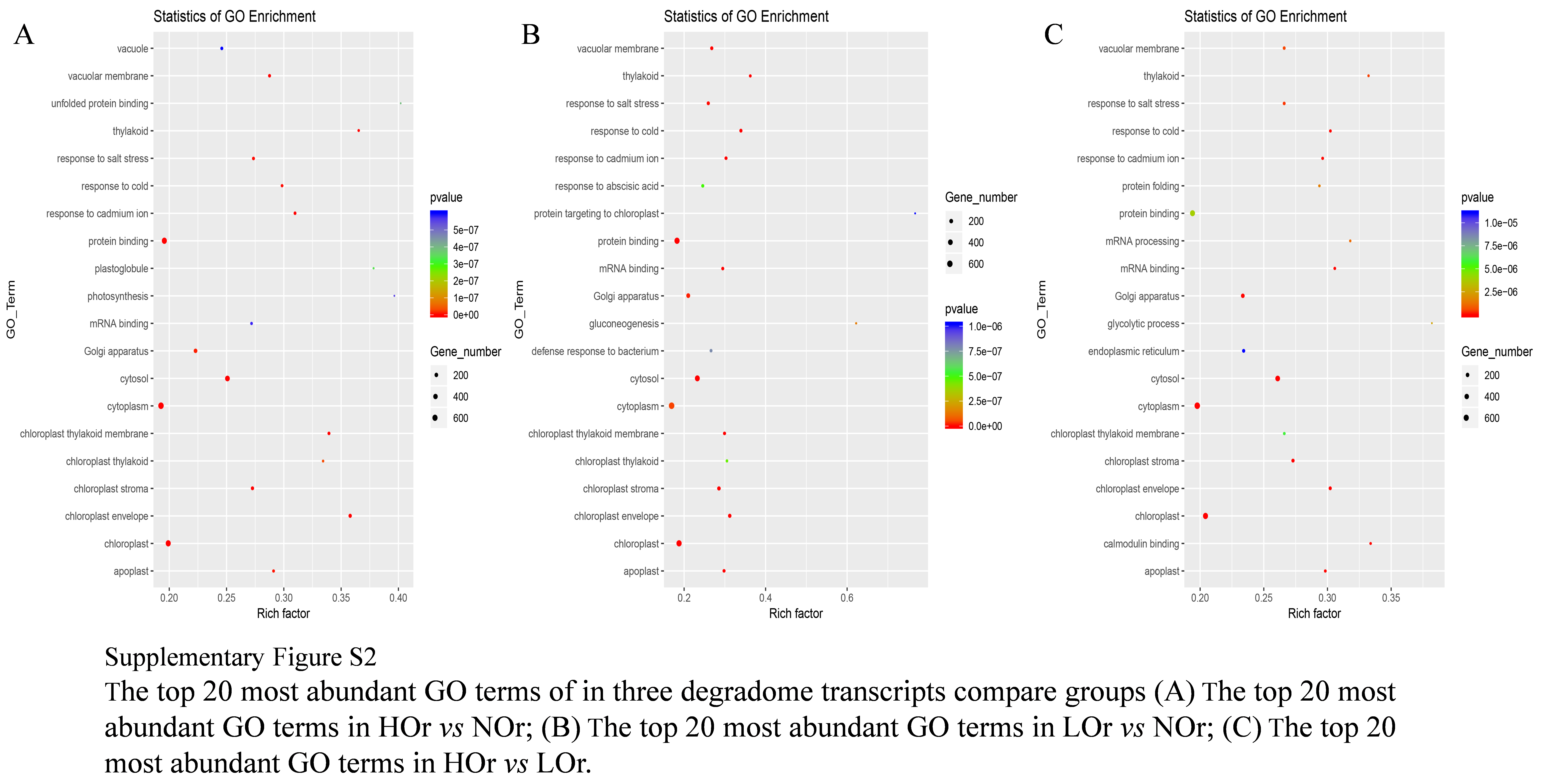

Supplement: Supplementary file 1 [file DataSheet1.zip › Supplementary Material/Supplementary Figure S2.tif]
